# Highly sensitive label-free bio-interfacial colorimetric sensor based on silk fibroin-gold nanocomposite for facile detection of chlorpyrifos pesticide

**DOI:** 10.1038/s41598-020-61130-y

**Published:** 2020-03-06

**Authors:** Pramod C. Mane, Manish D. Shinde, Sanjana Varma, Bhushan P. Chaudhari, Amanullah Fatehmulla, M. Shahabuddin, Dinesh P. Amalnerkar, Abdullah M. Aldhafiri, Ravindra D. Chaudhari

**Affiliations:** 1 P. G. Department of Zoology and Research Centre, Shri Shiv Chhatrapati College of Arts, Commerce and Science, Junnar, 410 502 India; 2 Centre for Materials for Electronics Technology, Panchwati, Off Pashan Road, Pune, 411 008 India; 3 Biochemical Sciences Division, CSIR-National Chemical Laboratory, Pashan, Pune, 411 008 India; 4 Department of Physics and Astronomy, College of Science, King Saud University, Riyadh, 11451 Saudi Arabia; 5 School of Mechanical Engineering, Sungkyunkwan University, Suwon, 440 746 South Korea

**Keywords:** Environmental monitoring, Spectrophotometry, Nanoparticles

## Abstract

Herein, the preparation of gold nanoparticles-silk fibroin (SF-AuNPs) dispersion and its label-free colorimetric detection of the organophosphate pesticide, namely chlorpyrifos, at ppb level are reported. The silk fibroin solution was extracted from *B. mori* silk after performing degumming, dissolving and dialysis steps. This fibroin solution was used for synthesis of gold nanoparticles *in-situ* without using any external reducing and capping agent. X-ray Diffractometry (XRD), Field Emission Transmission Electron Microscopy (FETEM) along with Surface Plasmon Resonance based optical evaluation confirmed generation of gold nanoparticles within SF matrix. The resultant SF-AuNPs dispersion exhibited rapid and excellent colorimetric pesticide sensing response even at 10 ppb concentration. Effect of additional parameters viz. pH, ionic concentration and interference from other pesticide samples was also studied. Notably, SF-AuNPs dispersion exhibited selective colorimetric pesticide sensing response which can be calibrated. Furthermore, this method was extended to various simulated real life samples such as tap water, soil and agricultural products including plant residues to successfully detect the presence of chlorpyrifos pesticide. The proposed colorimetric sensor system is facile yet effective and can be employed by novice rural population and expert researchers alike. It can be exploited as preliminary tool for label-free colorimetric chlorpyrifos pesticide sensing in water and agricultural products.

## Introduction

Pesticides act as toxic compounds to all living organisms and hence used to control the insects, weeds, nematodes and other various pests. The pesticides can be carcinogenic in nature, which can produce disorders in bone marrow and nervous system and can sometimes cause infertility and respiratory diseases. Organophosphates (OPs) are among the major pesticides that are largely used in agriculture owing to their relatively low persistence and high effectiveness in insect and pest eradication^1^. They are synthetic esters, amides, or thiol derivatives of the phosphoric, phosphonic, phosphorothioic, or phosphonothioic acids^2,3^ and are used all over the world to control agricultural, household and structural pests. They are usually considered as safe for agricultural applications due to their relatively faster degradation rates^4^. Over the past several years, organophosphate turned out to be an important parameter for water quality evaluation^5^. Pesticides in residual form can enter into humans through the food chain (fishes, raw vegetables, fruits and water)^6^. As insecticidal mechanism, OPs inhibit acetylcholine-esterase (AChE) activity in insects providing the pesticide action^4,7,8^. However, they can also affect the nervous system of other organisms as well as humans imparting toxicity and number of environmental and health issues associated with them have not only provoked the public concern but also research activities for finding remedies^6^. Some class of OPs is even considered as lethal weapons of mass destruction^9^. Despite claims of their fast degradation rates, literature data illustrated that the OPs persist in soils years after their application^10^.

The United State Environmental Protection Agency (USEPA) has specially published the report of OP risk assessments and has prescribed a maximum residue limit of OP in almost all agricultural products, for instance, the maximum residue limit of 10 ppb is allowed by the EPA in apple juice^11^. Although classical analytical methods (including gas chromatography, high performance liquid chromatography, capillary electrophoresis and mass spectrometry) have been effectively used for analysis of pesticides in contaminated samples, they impose certain limitations such as time-consuming sample preparation, process complexity and the requirement of expensive instrumentation and highly skilled personnel^12^.

Even though OPs have the short life time of decomposition, they persist and leach out in soil and environment which is the matter of huge concern. Hence, there is a need to develop the new moieties which can minimize all these risks and hazards. Besides, it is also highly desirable to develop quicker, cheapest and portable methods for pesticides sensing. Detection of pesticides at lower concentrations remains a challenge. Several methods like chromatography have been used for pesticide analysis. But such methods have certain drawbacks as they are time consuming, laborious and also require costly equipment and trained technicians. The development of facile biosensors for pesticide detection is a promising alternative^13^.

Among various sensing strategies, nanoparticles-based detection is desirable due to high extinction coefficient and specific optical properties of the nanoparticles^14^. Moreover, nanoparticles-based sensors can be advantageous in terms of rapid sensing, reliability, easy portability and minimum operational training^15^. In the past, colorimetric biosensors based on noble metal nanoparticles, namely gold and silver have been reported for the detection of bacteria, heavy metals, macromolecules and other analytes^15–24^. They have been also used for the detection of pesticides^25,26^. However, a lot of work is still required for establishing colorimetric bio-sensing for pesticide detection as versatile, simple and cheap tool appealing to mass population. One of the key aspects in this direction is to explore the synthesis of noble metal nanoparticles in a suitable matrix, preferably a biopolymer. Additionally, such biopolymer should be easily available, cheap and easy to process. In this context, silk is a wonder material; it is cheap and versatile as far as availability is concerned. Silk fibroin (SF), the biopolymer macromolecule component of silk, can be easily prepared and processed^27^. Silk fibroin, a multi-domain protein, has gained attention because of its notable properties of good mechanical strength, biocompatibility, minimal inflammatory, slow biodegradability and capability to reduce metal ions into neutral atoms^28^. There are some reports on the synthesis of gold and silver nanoparticles in SF matrix^27,29–32^. However, to the best of our knowledge, it has still not been explored as a colorimetric biosensor material for pesticide detection.

Herein, we report an acetylcholine-esterase (AChE) label-free bio-interfacial colorimetric method for the detection of organophosphate pesticide (viz. chlorpyrifos) in the aqueous solution. Our SF-AuNPs based colorimetric sensor can detect the OP concentration as low as 10 ppb. The methodology is very simple and can be used by rural population for preliminary detection of presence of pesticides in water or other commodities. This process will also reduce the workload of sophisticated instruments for analyzing large number of samples. Only the samples, which have been screened by such bio-interfacial colorimetric sensor, can be further analyzed by costly analytical instruments thereby offering savings in resources, cost and manpower.

## Results and Discussion

**Figure 1:**
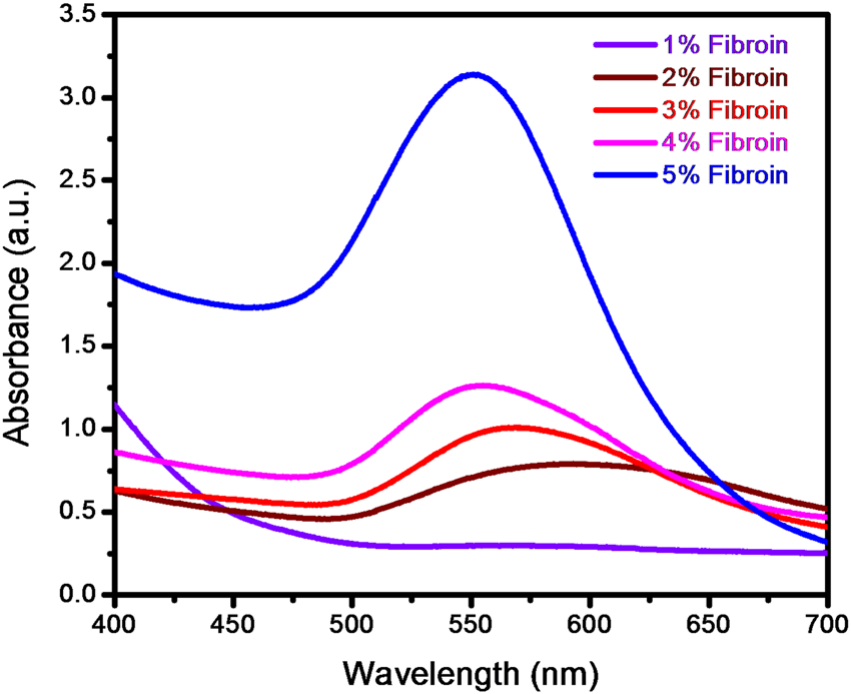
UV – Vis. spectra of gold nanoparticles synthesized from different percentage of silk fibroin.

Figure 1 exhibits the UV-Visible spectra of gold nanoparticles synthesized from 1% to 5% of silk fibroin. The results disclose that the sharp absorbance peak is observed in the vicinity of 535 nm in case of solution containing 5% fibroin which evidently points out the formation of gold nanoparticles. From the literature, it is noted that silk fibroin consists of 18 amino acids, including tyrosine. During the synthesis of SF – AuNPs, when the pH is adjusted to 9–10, the phenolic component of the tyrosine residue gets transformed into phenoxide anions which enhances the electron density in the п-п* transition of Tyrosine. This helps Au^3+^ to obtain stable state by exchanging an electron from the Tyrosine residue^33^.

The synthesized SF–AuNPs were stored at room temperature in the dark conditions. The stability of the AuNPs was checked after 9 months by UV-Visible spectroscopy which displayed similar absorbance peak. This result confirms that, the SF-AuNPs solution can be stored for longer duration and can be used whenever necessary^28^.

**Figure 2:**
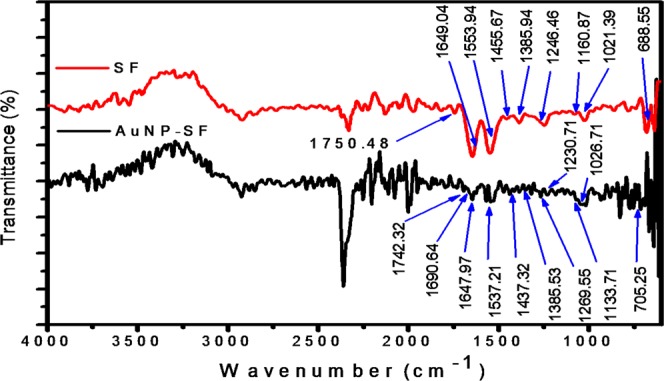
FTIR spectra of SF and SF-AuNP nanocomposite.

Figure 2 shows the ATR-FTIR spectra of SF and SF-AuNPs. The peaks in the range of 1600 cm^−1^ to 1700 cm^−1^, 1500 cm^−1^ to 1600cm^−1^,1224 cm^−1^ to 1260 cm^−1^ and 690 cm^−1^ can be assigned to amide I, amide II, amide III and amide IV region of protein, respectively. The characteristic peaks in these ranges indicate the presence of all these regions in both SF and SF-AuNPs except amide IV region in SF-AuNPs,^28,30,34,35^. The peak at 1649 cm^−1^ signifies -C=O stretching of amide I region and presence of random coil structure in SF. The peaks at 1649 cm^−1^ and 1553.9 cm^−1^ are blue shifted to 1647.9 cm^−1^and 1537 cm^−1^ in SF-AuNPs. Besides, the intensity of these peaks is also decreased which implies the formation of gold nanoparticles^36,37^. An extra peak at 1160.8 cm^−1^ is due to the pentoxide anions of the phenolic component of tyrosine residues, which helps in formation of AuNPs in accordance with the earlier mentioned mechanism. The absence of this particular peak in SF-AuNPs with simultaneous appearance of peak in the range 1725 cm^−1^ to 1745 cm^−1^ might correspond to formation of quinone by oxidation of -OH group of tyrosine molecules^28,34,38^. This observation extends additional support for the synthesis of gold nanoparticles within SF matrix.

**Figure 3:**
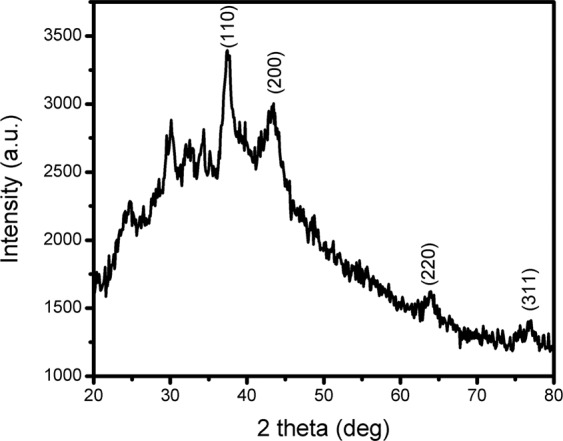
X-ray diffractogram of silk fibroin film containing gold nanoparticles.

Figure 3 shows the typical X-ray diffractogram of SF-AuNPs sample. The sample reveals four characteristic peaks corresponding to (110), (200), (220) and (311) planes of face centered cubic (FCC) crystal structure of gold matching with JCPDS File No 04-0784. Significant peak broadening is an indication of formation of nanoscale products. Additionally, the sample exhibits a broad hump engulfing almost all the crystalline peaks of the sample. Such hump may be attributable to amorphous phase of the silk fibroin while the crystalline peaks in the range of 20° to 35° may be due to crystalline coil like phase of the fibroin^39^. The absence of LiBr peaks in the XRD pattern (corresponding to the JCPDS File No. 74-1973) rules out the possibility of presence of LiBr as impurity phase which is used for dissolving silk fibroin and is removed during dialysis.

**Figure 4:**
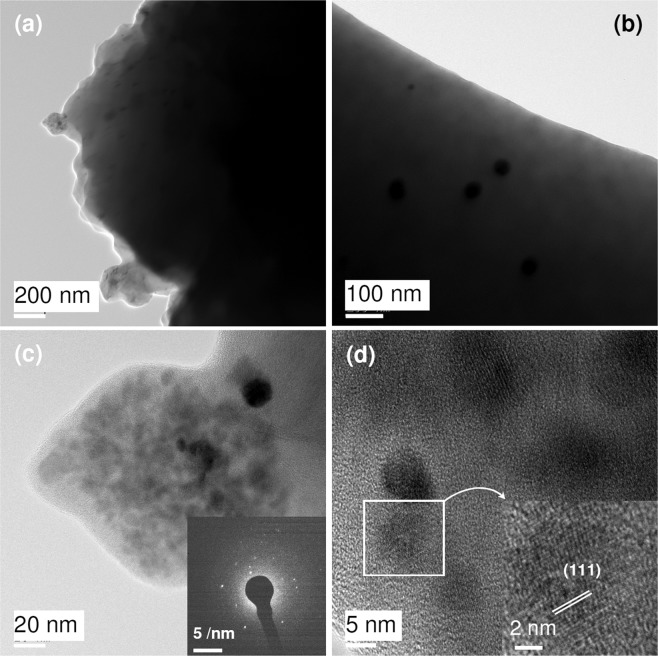
FETEM images of silk fibroin films containing gold nanoparticles at - (**a**) low, (**b**) intermediate, (**c**) high magnification and (**d**) lattice image. Inset of Fig. 2c,d represent SAED and lattice patterns, respectively.

Dynamic Light Scattering (DLS) is used to determine the hydrodynamic diameter and zeta potential of SF-AuNP. The average hydrodynamic diameter of SF-AuNPs is 34.8 nm (Table 1, Blank) with polydispersity index (PDI) of 0.379. The zeta potential represents the surface charge and stability of nanoparticles. In this study, the negative average zeta potential 29 mV is observed which indicates the stability of SF-AuNPs^28^. FETEM images of SF-AuNPs sample are shown in Fig. 4. The FETEM image reveals amorphous silk fibroin matrix containing gold nanoparticles of different nanoscale sizes (Fig. 4a). It is difficult to obtain the information on size and shape of the nanoparticles at thicker regions. However, at thinner edges of the films, gold nanoparticles exhibit bimodal size distribution. Bigger nanoparticles of size 20 nm to 60 nm are seen occasionally (Fig. 4b). However, bunches of clusters of nanoparticles appear to be distributed throughout the silk fibroin matrix (Fig. 4c). The size of nanoparticles varies in such bunches of clusters in the silk fibroin matrix. It ranges from 2 nm to 10 nm as noticed in Fig. 4c,d. Quite interestingly, each nanoparticle appears to have structural orientation in particular direction. A representative lattice fringe image of Au nanoparticle having orientation along (111) is shown in the inset of Fig. 4d. The irregular spot like SAED pattern instead of ring like pattern also supports this observation (inset of Fig. 4c).

**Table 1:** Increase in hydrodynamic diameter of SF-AuNPs in relation with concentration of chlorpyrifos pesticide as analyte.

Concentration of chlorpyrifos (ppb)	Hydrodynamic Diameter of SF-AuNPs (nm)
Blank	34.8
10	68.6
20	173
30	194
40	223
50	438

**Figure 5:**
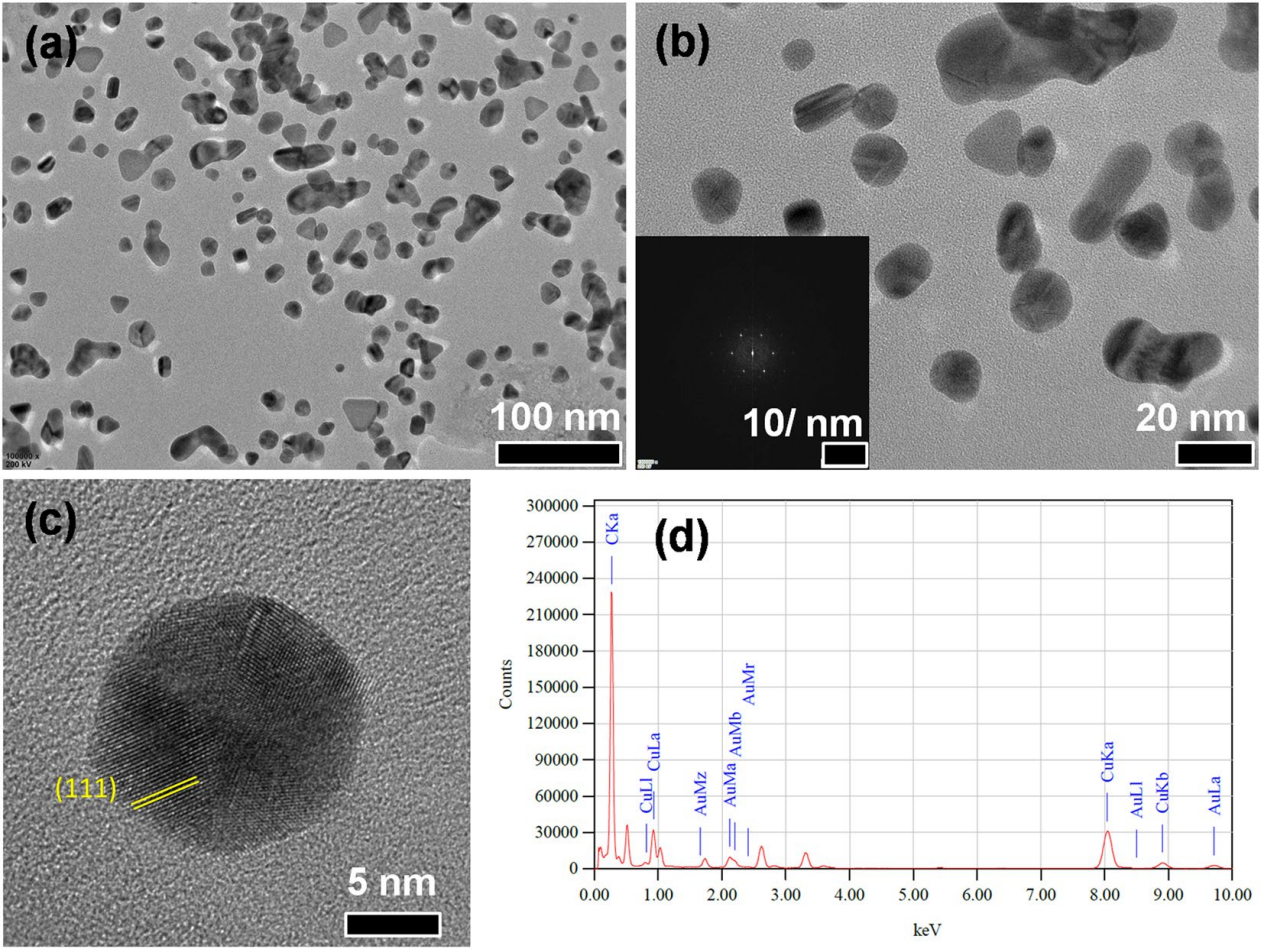
HR-TEM images of SF-AuNPs- (**a**) at 100 nm magnification, (**b**) at 20 nm magnification, (**c**) d- spacing of SF-AuNP and (**d**) EDAX spectrum of SF-AuNPs. Inset of (**b**) represents the FFT pattern.

Further to get Supplementary Information on size and shape of SF-AuNPs samples, HR-TEM analysis has been done. HR-TEM images in Fig. 5a,b disclose that there are irregularities in the shape of synthesized nanoparticles and the average size of AuNPs in SF matrix is 13 nm. The d-spacing value is 0.23 nm corresponding to (111) plane and well distinguished fringes are present (Fig. 5c). The Fast Fourier Transform (FFT) pattern of nanoparticles shown in the inset of Fig. 5b substantiates the crystalline nature of SF-AuNPs. The Energy Dispersive Analysis of X-rays (EDAX), as shown in Fig. 5d, proves the presence of Au element in the sample, eventually confirming the formation gold nanoparticles. The EDAX peaks of copper and carbon can originate from the TEM grid.

In our previous work, it has been illustrated that UV-visible spectrum of the silk fibroin solution containing gold nanoparticles exhibits a Surface Plasmon Resonance (SPR) peak in the vicinity of 520 nm, which is a characteristic peak for gold nanoparticles and, in turn, confirms formation of gold nanoparticles within the SF solution matrix^40^. The gold nanoparticles produced through biological process in our study exhibit ruby red color which appears due to excitation of localized surface plasmon vibration of the metal nanoparticles^40,41^. The SPR peak broadening towards higher wavelength side may be because of capping environment (proteins) and/or wide particle size distribution^42^.

### Pesticide detection

Chlorpyrifos (CPS) is an organophosphate pesticide used for crops, animals, buildings, and in other settings in order to kill a number of pests including insects and worms. The largest agricultural market for chlorpyrifos in terms of total pounds of active ingredient is corn. It is also used as an effective pesticide in case of soybeans, fruit and nut trees, Brussels sprouts, cranberries, broccoli, and cauliflower, as well as other raw crops. Non-agricultural uses include golf courses, turf, green houses, and non-structural wood treatment such as utility poles and fence posts. It is also registered for use as a mosquito adulticide^43^. But, the study revealed that, the exposure of chlorpyrifos affects brain development of children as it is a neurotoxic pesticide^44^. Hence, in the present study, we focused only on the chlorpyrifos for colorimetric detection.

**Figure 6:**
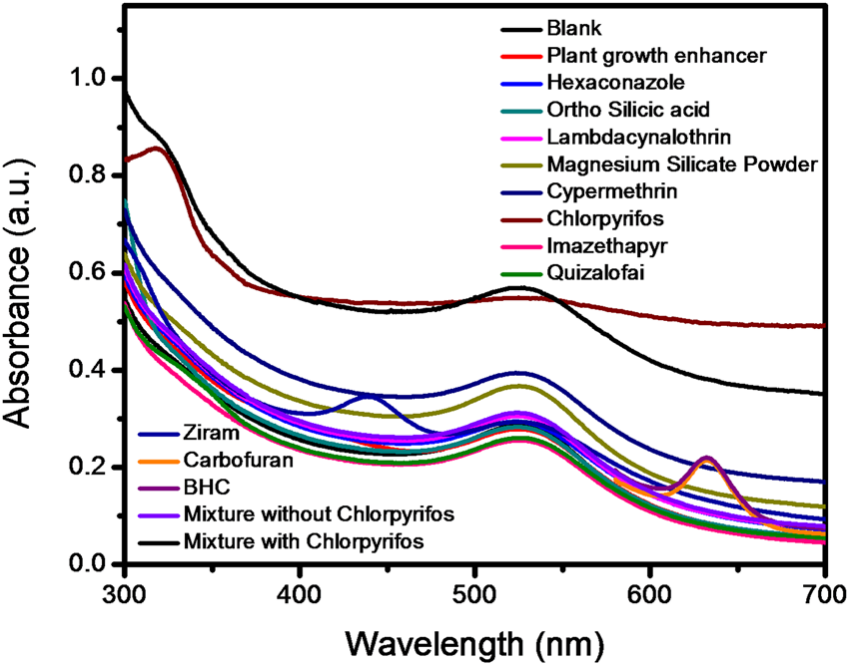
Selective bio-interfacial sensor for individual chlorpyrifos pesticide, as well as from the mixture of pesticides and other chemicals.

Conventional yet simple UV–Visible spectroscopy was employed to quantitatively ascertain the feasibility of colorimetric assay for qualitative naked-eye-based assertion. UV-visible absorbance spectra of various pesticide samples, namely benzene hexachloride (BHC), carbofuran, plant growth enhancer, hexaconazole, ortho silicic acid, lambdacynalothrin, magnesium silicate powder, cypermethrin, chlorpyrifos, imazethapry, quizalofai, ziram, mixture without chlorpyrifos and mixture with chlorpyrifos are shown in Fig. 6. The absorbance spectra corresponding to all above individual pesticides, except chlorpyrifos, did not display any significant increase in the intensity of the Au-NP SPR peak as compared to the blank (control) sample (Fig. 6). However, a significant increase in the intensity of SPR absorbance peak is noticed in both the cases when individual chlorpyrifos and its mixtures with other pesticides are studied. Such a significant increase in the intensity is the clear indication of the selective sensor response of the colorimetric sensor towards chlorpyrifos which is an organophosphate. Therefore, most of the further pesticide detection study was confined to chlorpyrifos. Gold nanoparticles can be used as a colorimetric sensor to detect pollutants qualitatively and quantitatively from the water. The colorimetric sensor of gold nanoparticles is mainly based on the inter-particle Surface Plasmon Coupling which results in macroscopically visible color change^45^.

Generally, the color changes are highly sensitive to aggregation state of gold nanoparticles size, shape and capping environment and this can be confirmed by absorbance band-shift in the visible region of the electromagnetic spectrum^46^. This may be due to the aggregation of nanoparticles by the adsorption of cationic species on the surface^47^.

Several researchers developed gold nanoparticles based colorimetric sensor for the detection of chemical species including heavy metal ions, pesticides, micro-organisms etc.^15,48,49^. Some authors reported that gold nanoparticles can detect the pesticides at ppm level by direct binding of pesticides to the gold nanoparticles surface through the –S=O (in endosulfan), –P=S (in chlorpyrifos), -P=O (organophosphates) bonds^48^. Similarly, Newman *et al*., developed sensor to measure organophosphate by using gold nanoparticle functionalized silica^50^. Some authors also developed a colorimetric sensor by using gold nanoparticles for detection of organophosphate^51^.

**Figure 7:**
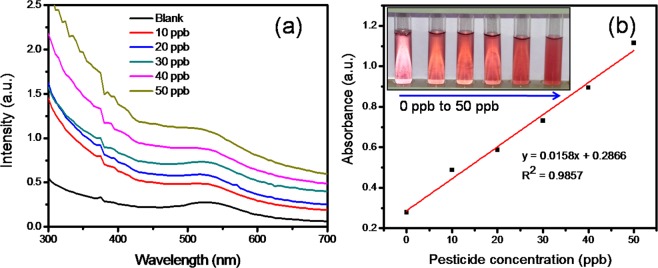
UV-vis spectra corresponding to different concentrations of pesticide solution injected with - (**a**) SF-AuNP solution and (**b**) calibration curve associated with chlorpyrifos–SF-AuNP solutions after incubation for 30 min. Inset of Fig. 7b shows the digital photographs of blank SF-AuNP solution and SF-AuNP solution mixed with different concentrations of chlorpyrifos pesticide.

UV–vis spectra corresponding to different concentrations of pesticide solution injected with SF-AuNP solutions after incubation for 30 min are provided in Fig. 7.

At this juncture, it may be noted that the current pesticide sensor based on SF-AuNPs solution (Fig. 7a) exhibits gradual increase in the intensity and broadening of SPR peak at 535 nm with the addition of chlorpyrifos pesticide solution in the concentration range of 10 ppb to 50 ppb. The broadening in the peak is ascribable to the increase in agglomeration of nanoparticles. This is the clear qualitative pesticide sensing response of SF-AuNPs solution. The corresponding changes in the color of the solution which can be detected with the naked eye are shown in the inset of the Fig. 7b which implies the simplicity of our colorimetric sensor. A more quantitative inference about the sensor response can be obtained by plotting the calibration curve (at 535 nm) as shown in Fig. 7b. The curve demonstrates a linear response in the desired concentration region of 10 ppb to 50 ppb with R^2^ = 0.9857.

**Figure 8:**
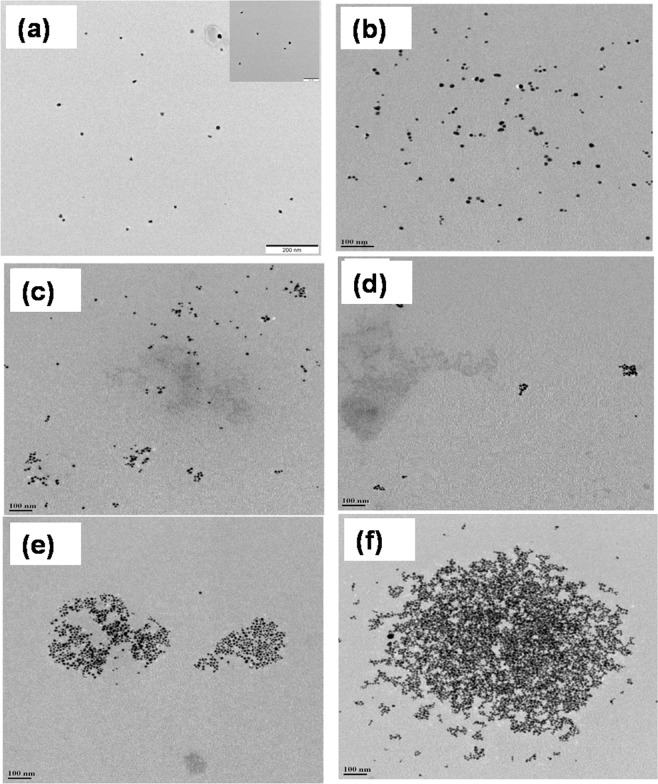
TEM images of agglomerated SF-AuNPs after treating with various concentrations of chlorpyrifos pesticide analyte – (**a**) control, (**b**) 10 ppb, (**c**) 20 ppb, (**d**) 30 ppb, (**e**) 40 ppb and (**f**) 50 ppb.

Further confirmation of agglomeration is done by both DLS and TEM studies. The hydrodynamic diameter of SF-AuNPs increases as the concentration of chlorpyrifos increases as shown in Table 1. From the relevant TEM analysis, it is confirmed that the gold nanoparticles tendto agglomerate (Fig. 8). The silk fibroin provides requisite stability to the gold nanoparticles thereby keeping the nanoparticles well-dispersed upon linking which is attributable to its random coil structure. Addition of the target pesticide, i.e. chlorpyrifos causes a rigid conformation in the silk fibroin structure which culminates in the aggregation of the gold nanoparticles ultimately changing the color of the nanocomposite dispersion which can be observed even with the naked eye.

**Figure 9:**
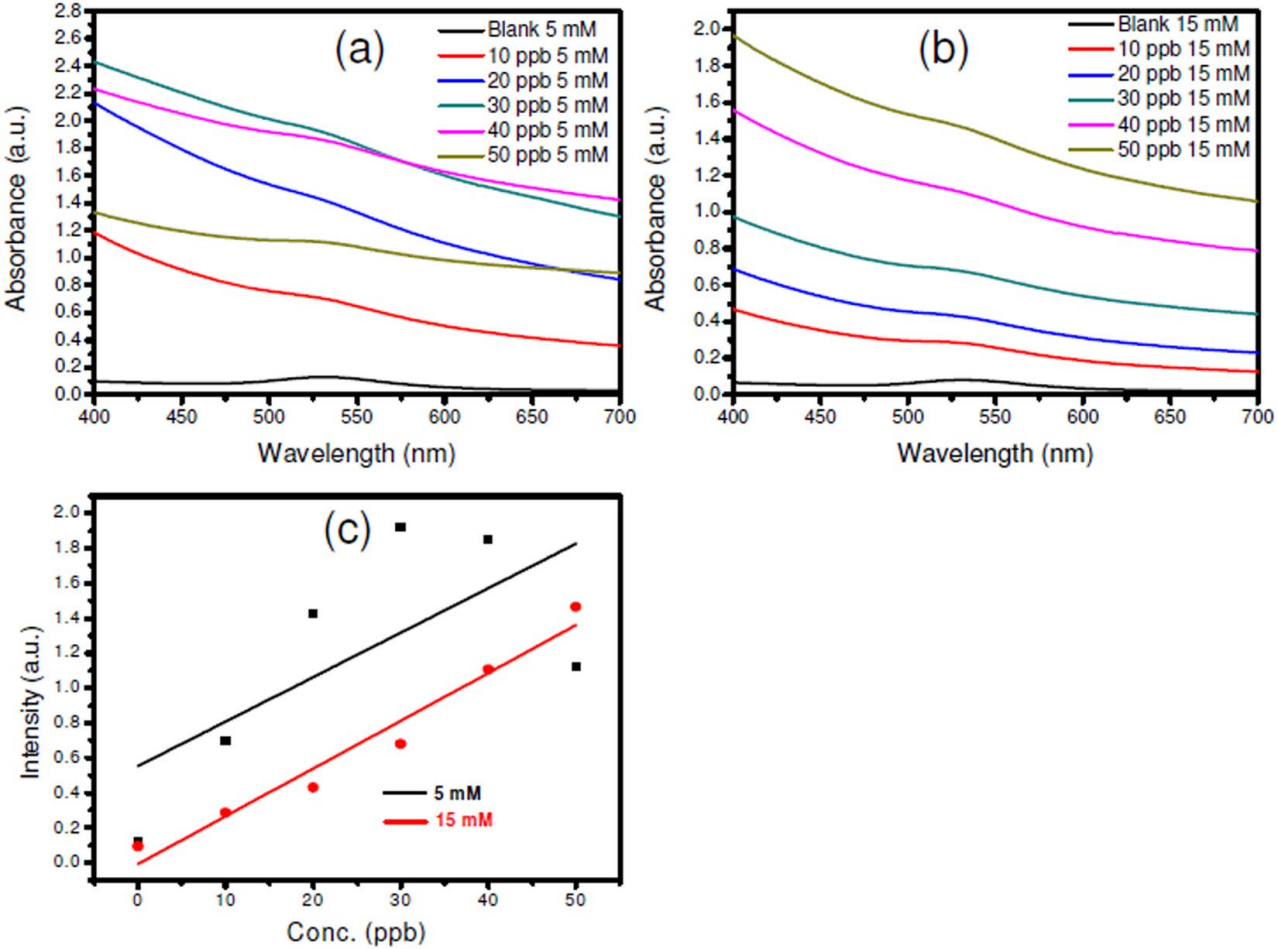
Effects of ion concentration: (**a**) 5 mM and (**b**) 15 mM (NaCl), on chlorpyrifos bio-interfacial sensor. Figure (**c**) is corresponding linear fit for Intensity vs. Concentration (in ppb) for both the concentrations of NaCl.

The study on effects of different ionic strength levels (0, 5 mM and 15 mM of NaCl) on chlorpyrifos sensing performance of SF-AuNP nanocomposite solution (at various concentrations) is shown in Fig. 9. It can be observed that the ionic strength level clearly affects the sensor behaviour. An increase in the absorbance intensity of the SPR peak position is clearly observed. However, this change is not consistent in case of lower molar concentration (5 mM) of NaCl (Fig. 9a). However, a systematic increase in the absorbance intensity is noted at higher (15 mM) concentration of NaCl. Increase in the range of intensity values is also noticed as compared to pure samples (see Fig. 9b) which may be helpful to resolve the absorbance intensity peak values in better way, thereby, increasing the accuracy of the sensing. The colorimetric pesticide sensor prepared using 15 mM NaCl has exhibited fairly linear sensing behaviour as compared to its counterpart prepared at 5 mM NaCl concentration (Fig. 9c).

**Figure 10:**
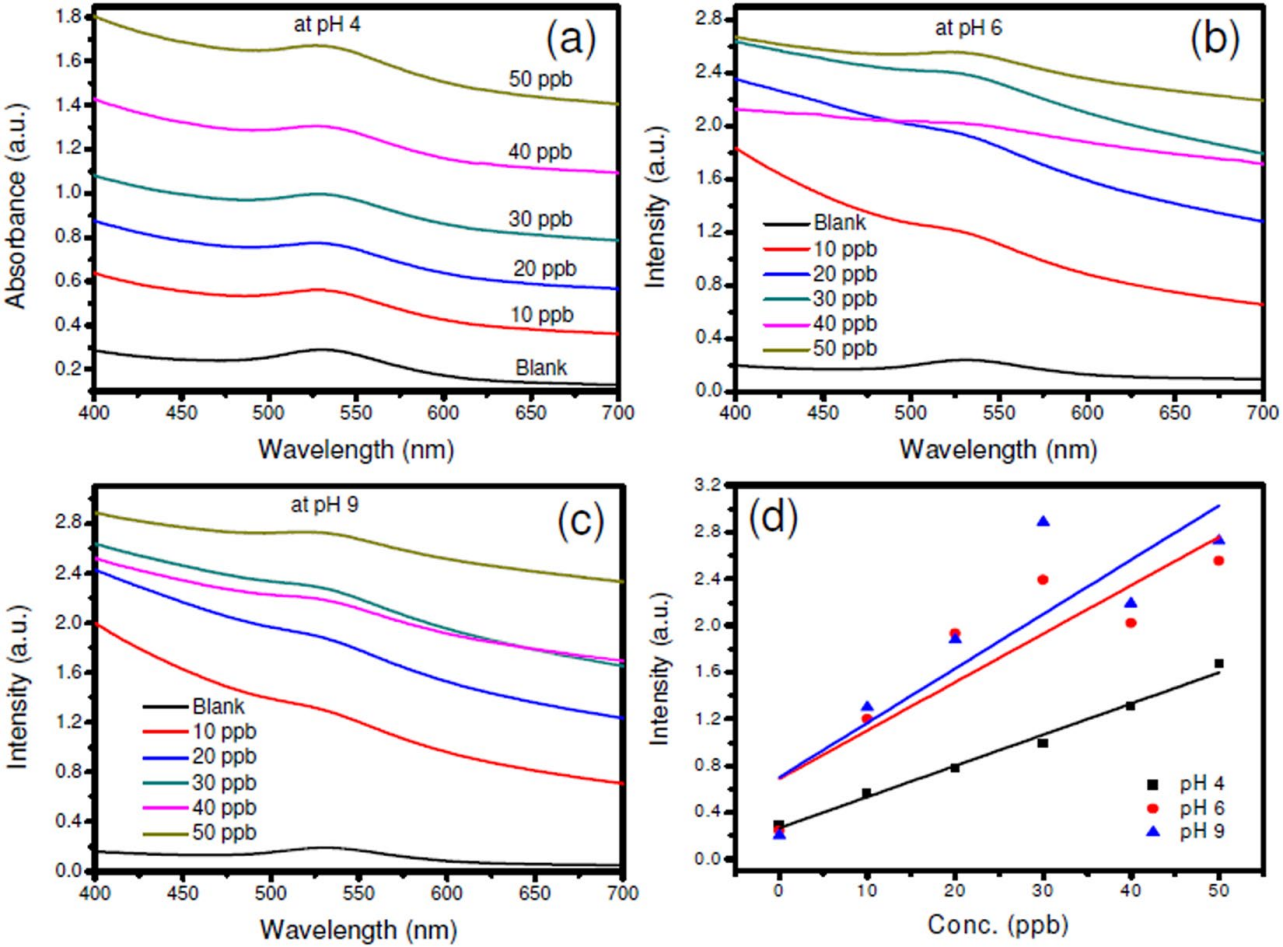
Effects of pH on chlorpyrifos sensor: (**a**) pH 4, (**b**) pH 6 and (**c**) pH 9. Figure (**d**) is corresponding linear fit for Intensity Vs. Concentration (in ppb) at pH values of 4, 6 and 9.

Significant effect of pH change on AuNP-based colorimetric sensors has been reported^52^. In the present case, under acidic conditions (pH ~ 4), an increase in the intensity of the SPR peak at 540 nm has been observed with the change in pesticide concentration (Fig. 10a). At higher pH values of 6 and 9 also, an increase in the intensity values of the absorbance at various pesticide concentrations is noticed (Fig. 10b,c). Thus, it can be noted that the current bio-interfacial sensor can be used under different pH conditions. However, it has exhibited linear behaviour for the change in the pesticide concentration at pH 4 as inferred from Fig. 10(d).

**Figure 11:**
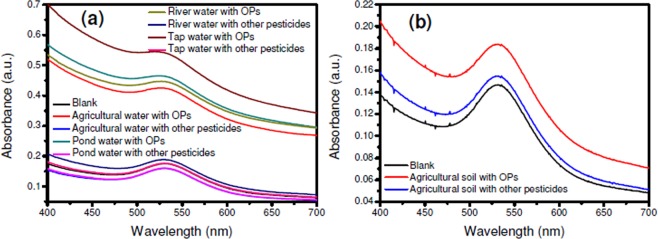
Real sample analysis of SF-AuNP nanocomposite solution based pesticide sensor towards–(**a**) water and (**b**) soil samples.

The colorimetric sensor response analysis of SF-AuNP nanocomposite solution based chlorpyrifos sensor towards the real water and soil samples in terms of change in the intensity of the SPR absorbance peak is shown in Fig. 11. When agriculture water, pond water, river water and tap water were spiked with mixture of other pesticides, notable change in the intensity of the SPR peak was not observed (Fig. 11a). However, when the similar water samples from the same sources were spiked with organophosphate (chlorpyrifos) and tested, a significant increase in the intensity of the SPR peak is observed. When agricultural soil sample spiked with mixture of other pesticides was tested as against chlorpyrifos towards SF-AuNPs nanocomposite solution, the similar trend in insignificant change in the SPR peak intensity value was noted. However, in this case, the difference in the intensity value was only 0.04 a.u.

**Figure 12:**
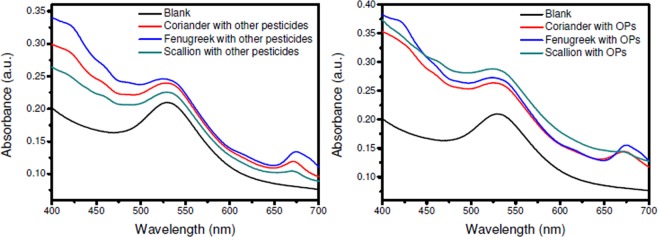
Real sample analysis for: (**a**) vegetable samples sprayed with mixture of BHC and Carbofuran, (**b**) vegetable samples sprayed with organophosphate (Chlorpyrifos).

In the third study on real samples, when vegetables like coriander, fenugreek and scallion were tested against a mixture of pesticides (Fig. 12a) using the SF-AuNP nanocomposite solution based pesticide sensor, slight increase in the intensity of the SPR peak as compared to the blank was observed. However, when tested against chlorpyrifos, slightly better increase in the intensity (with only 0.02 to 0.08 a.u.) was noted (Fig. 12b).

In United States, 38 major rivers were monitored to find out the presence of pesticide concentrations. In such elaborate study, it was found that, the river water contains eleven major pesticides including chlorpyrifos^53^. The global database on pesticide usage revealed that 13 African, 11 Caribbean & Central American, 24 European, 6 South American and 17 Asian countries use average of 1145, 4342, 10013, 13404 and 29554 metric tonnes of organophosphate. Such a huge use of organophosphates affects human health as well as fish and other aquatic life particularly in developing countries^54^. In California alone, around 927 metric tonnes of chlorpyrifos was used during 1999^55^. Previously, the sensor was developed for the detection of chlorpyrifos pesticide from water samples^56^.

The present investigation reports a very simple, easy to perform, rapid, label-free and cost effective method to detect chlorpyrifos, a widely used pesticide. Different methods for detection of pesticides are reported by several researchers. These methods include use of aptamers, Raman spectroscopy, fluorescent sensors etc^57–61^. But, such studies demand modification of nanoparticles with aptamers, optimization of reaction conditions, costly instruments involving prescribed sample preparations which are time consuming.

**Figure 13:**
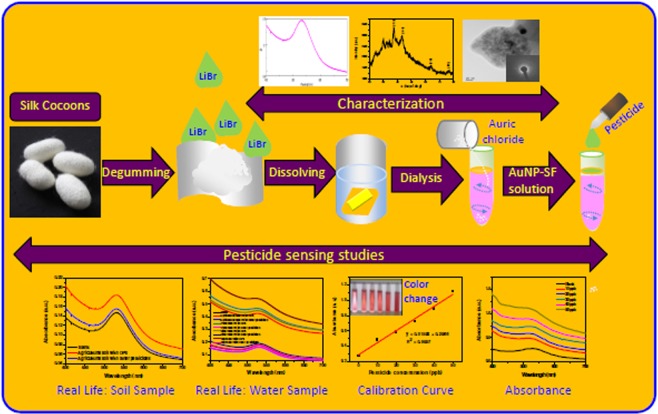
Schematic illustration of the preparation and pesticide sensing of silk fibroin-Au bio-nano-composite dispersion.

This study exhibited that the synthesized gold nanoparticles can be successfully used for the determination of chlorpyrifos pesticides from the water samples. Nonetheless, it was noted that such nanoparticles do not show any change in color when mixed with various concentrations of chlorpyrifos in absence of NaCl solution. When 10 M NaCl solution was mixed with the mixture of nanoparticles and pesticides, it tends to show change in color. Thus, chlorpyrifos can be selectively detected even from the real life samples of soil, water and vegetables by using the SF-AuNPs nanocomposite solution based pesticide sensor at the primary level. However, the sensitivity of the sensor is more in case of water based samples as compared to the soil and vegetables ones. The overall schematic illustration of the preparation and pesticide sensing by silk fibroin-Au bio-nanocomposite dispersion is provided in Fig. 13.

## Experimental

### Preparation of aqueous silk fibroin solution and synthesis of Au-nanoparticles

The process involves two steps, namely preparation of silk fibroin (SF) solution and *in-situ* synthesis of gold nanoparticles in SF solution. The pure silk fibroin was produced by following the standard protocol^62^. Initially, dried and cut pieces of *B. mori* silk cocoons were degummed by boiling treatment in aqueous solution of 0.02 M sodium carbonate. Fibroin solution was then generated by dissolving degummed silk fibers in 9.3 M LiBr solution at 70 °C for 2½ hours. The resultant fibroin solution was dialyzed in a cellulose membrane based dialyzed cassette (Thermo Scientific, Molecular Weight 10000 KD) against deionized water for 3 days. The resultant dialyzed solution containing 5% fibroin was used for the synthesis of gold nanoparticles by dissolving 0.5 mM auric chloride (HAuClO_4_) in 10 ml of dialyzed fibroin. The flask was incubated at room temperature in dark condition until its color is changed to ruby red. The same experiment was carried out with different concentrations of silk fibroin ranging from 1 to 5%.

### Physico-chemical characterization of SF-Au NPnanocomposite dispersion

ATR-FTIR (Bruker, Tensor 27) with detection range of 4000 cm^−1^ to 600 cm^−1^ was used for functional group analysis. X-ray diffractometry of the dried film of SF-AuNP nanocomposite was carried out by using X-ray diffractometer (Bruker, D8, ADVANCE, Germany) with Ni-filtered CuK_α_ radiation (λ = 1.54 Å). For this purpose, the film was drop-cast on a glass slide and dried under ambient conditions overnight. The particle size distribution, surface charge and stability of SF-AuNPs were determined by DLS (Malvern, Zetasizer Pro). Fine-scale microstructural evaluation of the SF-AuNP nanocomposite sample was accomplished by using FETEM (JEOL, USA, JEM ARM 200 F), HR-TEM (JEM F-200, URP) and TEM T-20 (FEI Technai, G2 system). For FETEM, TEM and HR-TEM analysis, the test sample was drop-cast on carbon coated TEM grid and dried overnight under ambient conditions in lamellar air flow. The optical property of the as-prepared solution mixture was studied by using UV –Vis Spectrophotometer (JASCO, V-770).

### Selective detection of pesticides

To carry out the tests for selective detection of pesticides in water samples, different pesticides, such as benzene hexachloride (BHC), carbofuran, plant growth enhancer, hexaconazole, ortho silicic acid, lambdacynalothrin, magnesium silicate powder, cypermethrin, chlorpyrifos, imazethapry, quizalofai, ziram, mixture without chlorpyrifos and mixture with chlorpyrifos were tested. In a typical experiment, 1 ml of standard solution of pesticides (1 µg/ml) was taken into 5 ml glass vial and mixed with 1 ml of silk fibroin – gold nanocomposite dispersion. The resultant solution mixture was incubated at room temperature for 5 minutes. Subsequently, the change in the intensity of the color and the localized surface plasmon resonance (LSPR) absorption band of the sample solutions were monitored by UV – VIS Spectrophotometer in the wavelength range of 300 to 700 nm. The pesticide which exhibited the highest selectivity was used for further pesticide sensing studies.

### Pesticide sensing activity

The stock solution of chlorpyrifos pesticide was prepared by diluting the pesticide to make the stock solution of 10 ppm. To carry out the sensing experiment, the standard solutions corresponding to concentrations *viz*., 10 ppb, 20 ppb, 30 ppb, 40 ppb and 50 ppb were made from the stock solution. An aliquot of OP’s standard solution/water/food sample was taken into 5 ml of glass bottle containing a mixture of 0.2 ml buffer and 0.3 ml of acetone and the volume was adjusted to 1 ml with milli Q water. To this mixture, 1 ml of SF-AuNPs dispersion was added and the resultant mixture was subjected to incubation at room temperature for 5 minutes. The change in the intensity of the color and the LSPR absorption band of the specimen mixtures were subsequently monitored by UV – VIS Spectrophotometer (JASCO, V-770).

### Effects of ionic strength and pH on detection of chlorpyrifos

Sensing study in the presence of ionic compound and at different pH is also important to validate the applicability of the sensor under different conditions^63^. We studied the effects of different ionic strength levels and various pH values on bio-interfacial sensing of pesticide. For this purpose, three ionic strength levels (0, 5, 15 mM NaCl) and three pH levels (4, 6 and 9) were selected. To carry out the intended study, various amounts of NaCl and HCl or NaOH solutions were added to the different concentrations of chlorpyrifos and milli Q water as a blank. To this mixture, SF-AuNPs dispersion was added and the resultant mixture was incubated for 5 minutes at room temperature until AuNPs reach the equilibrium before recording the spectra. All analysis was conducted in triplicate.

### Real sample analysis

In order to check the feasibility and applicability of the proposed SF-AuNPs based sensor in real samples, the detection of chlorpyrifos residues was carried out in irrigated agricultural water, river water, pond water and some crop/vegetable samples^64–66^. The water samples were filtered through filter paper, diluted 5 fold with milli Q water and then spiked with chlorpyrifos and other pesticides. To conduct the study on crops, five grams of crop samples were ground to the fine powder, spiked with chlorpyrifos and mixture of other pesticides. The samples were mixed with a mixture of 10 ml acetonitrile and 2 ml of milli Q water, sonicated for 30 minutes and finally centrifuged (Remi C – 24 Plus) at 4000 rpm for 10 minutes. The obtained supernatant was evaporated at 40 °C and the dried residue was diluted with 2 ml of ethanol. Finally, the analysis was performed on all the samples of water and crops in triplicate by following the same procedure implemented for chlorpyrifos detection.

## Conclusions

In modern nanotechnology, biomaterial enabled synthesis of nanoparticles is an important activity which is presently receiving prime attention. The present study deals with the synthesis of gold nanoparticles by using aqueous fibroin solution obtained from the raw silk of *Bombyx mori*. We have successfully demonstrated that such silk fibroin-gold nanocomposite solution can be used for label-free, rapid, selective, sensitive and economic colorimetric detection of chlorpyrifos pesticide. Quantitatively, the stated bio-nanocomposite system can detect the pesticide concentration as low as 10 ppb and can linearly detect even a 10 ppb change in the concentration with a correlation coefficient of 0.9984. The current bio-interfacial sensor was also tested for real life water, soil and vegetable samples. Curiously, it is observed that its sensitivity is more in case of water based samples as compared to the soil and vegetables ones. Such colorimetric pesticide sensor is easy to prepare and can be utilized by rural population for primary detection of the presence of harmful pesticides in the water reservoirs, soil, fruit and vegetable samples, etc. By making suitable changes like introduction of functional groups to bind gold nanoparticles, use of other metal nanoparticles, etc, the present approach can be further extended for more effective detection of other pesticides.
